# A Proteomic Analysis of Seed Development in *Brassica campestri* L

**DOI:** 10.1371/journal.pone.0050290

**Published:** 2012-11-26

**Authors:** Wenlan Li, Yi Gao, Hong Xu, Yu Zhang, Jianbo Wang

**Affiliations:** 1 State Key Laboratory of Hybrid Rice, College of Life Sciences, Wuhan University, Wuhan, China; 2 College of Chemistry and Bioengineering, Guilin University of Technology, Guilin, China; University of Nottingham, United Kingdom

## Abstract

To gain insights into the protein dynamics during seed development, a proteomic study on the developing *Brassica campestri L.* seeds with embryos in different embryogenesis stages was carried out. The seed proteins at 10, 16, 20, 25 and 35 DAP (days after pollination), respectively, were separated using two-dimensional gel electrophoresis and identities of 209 spots with altered abundance were determined by matrix-assisted laser desorption ionization time-of-flight/time-of-flight mass spectrometry (MALDI-TOF/TOF MS). These proteins were classified into 16 groups according to their functions. The most abundant proteins were related to primary metabolism, indicating the heavy demand of materials for rapid embryo growth. Besides, the high amount of proteins involved in protein processing and destination indicated importance of protein renewal during seed development. The remaining were those participated in oxidation/detoxification, energy, defense, transcription, protein synthesis, transporter, cell structure, signal transduction, secondary metabolism, transposition, DNA repair, storage and so on. Protein abundance profiles of each functional class were generated and hierarchical cluster analysis established 8 groups of dynamic patterns. Our results revealed novel characters of protein dynamics in seed development in *Brassica campestri L.* and provided valuable information about the complex process of seed development in plants.

## Introduction

Plant seed is an important organ that is evolutionarily advantageous for plant survival and contributes so much to animal and human life [1]. Seed development goes through three overlapping phases, i.e. morphogenesis, seed filling and seed desiccation, which involve coordinated growth of three seed components, seed coat, endosperm and embryo [2].

Seed development involves highly dynamic processes of cell division, differentiation, growth, pattern formation and macromolecule production [3], [4], elucidating the underlying mechanisms will provide insight into the complex system coordinating plant development and metabolism. In recent years, genetic and molecular analyses have identified critical players in the process of seed development [1]. DNA microarray and RNA-seq technique are also advantageous by large-scale genome-wide study at the mRNA level [5]–[7]. However, mRNA level doesn’t always reflect protein abundance [8], and genomic tools can’t provide precise information on protein levels [9], limiting our understanding on those metabolic and molecular networks. Proteomics provides more powerful tool to understand the complex protein dynamics and the underlying regulatory mechanisms during seed development [10]–[12]. By examining temporal patterns and simultaneous changes in protein accumulation, extensive proteomic studies have been carried out in legumes [13], [14], Arabidopsis [15], [16], rapeseed [17], [18], rice [19], wheat [20], [21] and many other species [10] to profile protein dynamics during seed development. The most popular proteins are those participating in central metabolism, followed by those related to cellular structure, and many previously unknown proteins are indicated important roles in embryo development [12]. In addition, proteome studies also reveal some important characters of seed proteins. For example, a proteome study on *Medicago truncatula* reveals a remarkable compartmentalization of enzymes involved in methionine biosynthesis between the seed tissues, therefore regulating the availability of sulfur-containing amino acids for embryo protein synthesis during seed filling [22]; in tomato seed, the most abundant proteins in both the embryo and endosperm were found to be seed storage proteins, such as legumins, vicilins and albumin [23]. These proteomic applications have greatly expanded our knowledge on seed development.

Plant embryo development, also known as embryogenesis, defines an important development process in higher plant life cycle [24]. Embryo development starts from a double fertilization event in which two sperm nuclei fuse with the egg cell and central cell nuclei respectively, then the zygote undergoes a series of cell divisions and differentiation events to initiate embryo development, going through a globular embryo stage, then a heart-stage, a torpedo-stage and a bended-cotyledon-stage embryo consecutively to produce the mature embryo [25]–[27]. Therefore, embryogenesis covers part of the processes of morphogenesis and seed filling during seed development. There are gaps in our understanding on the complete seed development process, as current proteomic studies mainly focus on the protein dynamics during the seed filling or seed dessication. A systematic view of the seed development process encompassing complete embryo development stages is necessary for integrity of our knowledge of full seed development. This is especially meaningful for most dicot plants, because in the mature seed of different species, the relative content of endosperm and embryo is variable. The embryo in dicots is normally the major part of the mature seed, such as in species of *Arabidopsis thaliana* and *Brassica napus*, and the endosperm is almost completely absent in the mature seed, whereas in monocts such as wheat, maize, and rice, endosperm tissues possess the majority of the whole seed mass.

To this end, oilseed (*Brassica campestri* L.) takes its advantage for its relatively larger embryo compared to the model plant Arabidopsis and ease to be accurately differentiated from embryo developmental stages [28]. *B. campestri* belongs to the mustard family (Brassicaceae), and like most dicotyledonous plants, its embryo development goes through morphologically defined globular, heart, torpedo, and bended cotyledon stages to produce the mature embryo [25], [26], [29]. Here, using *B. campestri* seeds with embryos in five sequential development stages of embryogenesis, we carried out a proteomic study on protein dynamics aiming at understanding seed development of oilseed.

## Results

### High-resolution Proteomes of the Developing *B. campestri* Seeds

**Figure 1:**
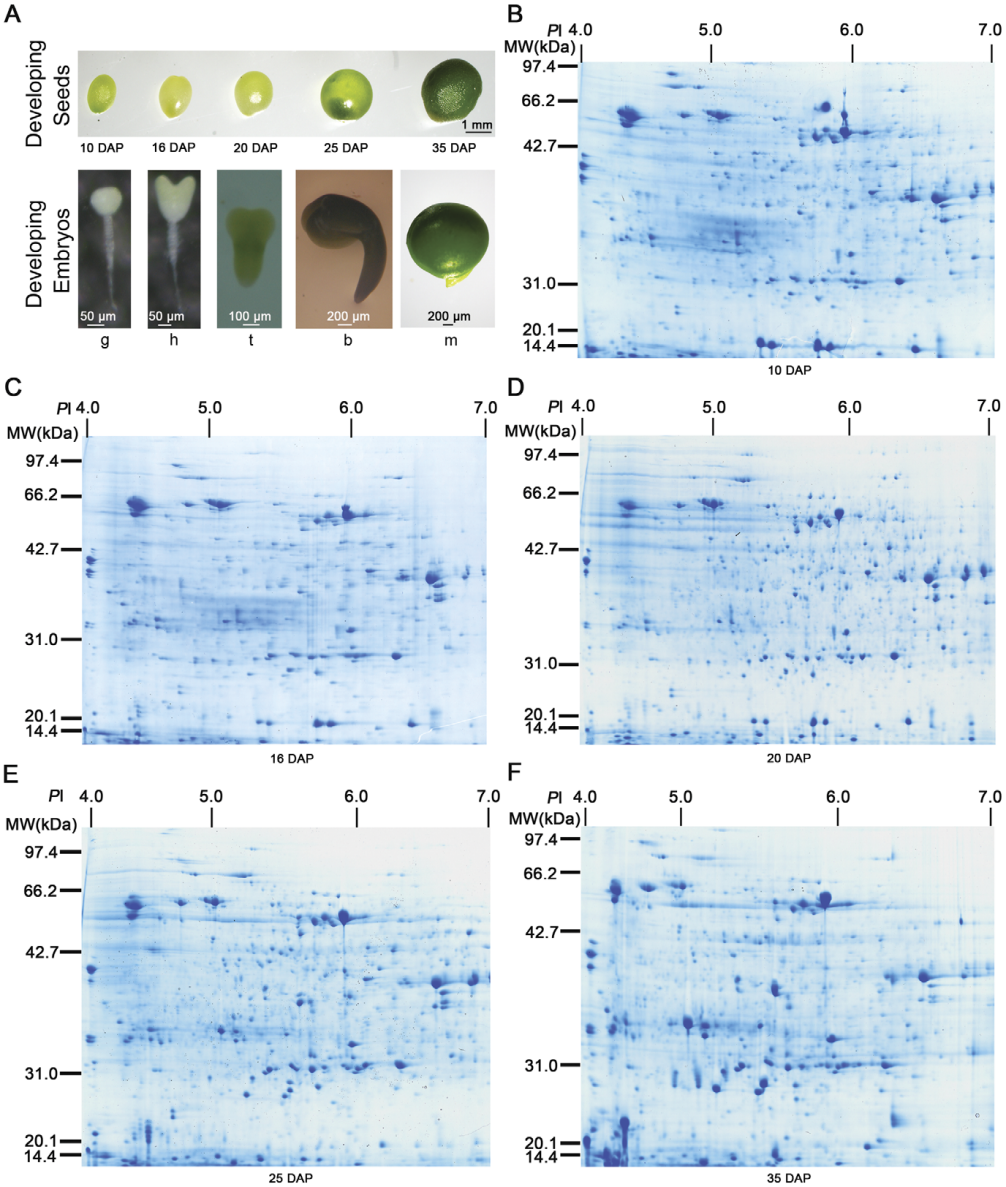
Two-dimensional gel electrophoresis analysis of proteins isolated from developing *Brassica campestri* L. seeds. Seed samples were collected at 10 (**B**), 16 (**C**), 20 (**D**), 25 (**E**) and 35 (**F**) days after pollination (DAP) respectively (**A**). Seed development were monitored by observing their embryos that were at globular stage (g), heart stage (h), torpedo stage (t), bended cotyledon stage (b), and mature embryo stage (m), respectively (**A**). Total seed proteins were separated by 2-DGE with IEF (pH 4–7) and detected by Coomassie Brilliant Blue.

To isolate proteins of seeds at different stages, developing *B. campestri* seeds were harvested at precisely 10, 16, 20, 25 and 35 DAP when their embryos were in the globular embryo stage, heart stage, torpedo stage, bended-cotyledon stage and C-shaped mature embryo, respectively (Figure 1). Then the whole proteins were resolved and detected using high-resolution two-dimensional electrophoresis (2-DE) followed by colloidal Coomassic brilliant blue staining. Initial analyses were performed with immobilized pH gradient (IPG) strips that ranged from pH 3 to 10. It was observed that the region from pH 4 to 7 was a highly dense area on the proteome map, so analyses with pH 4 to 7 IPG strips were further performed to attain high resolution proteome maps. The 2-DE maps showed a highly dynamic proteome during *B. campestris* seed development (Figure 1). Using the ImageMaster 2D Platinum software 6.0 (GE Healthcare), more than 800 CBB R250-stained protein spots were reproducibly detected from at least three independent 2-D gels, suggesting they were involved in the seed development.

### Identification of Dynamically Accumulated Seed Proteins of *B. campestri*

**Figure 2:**
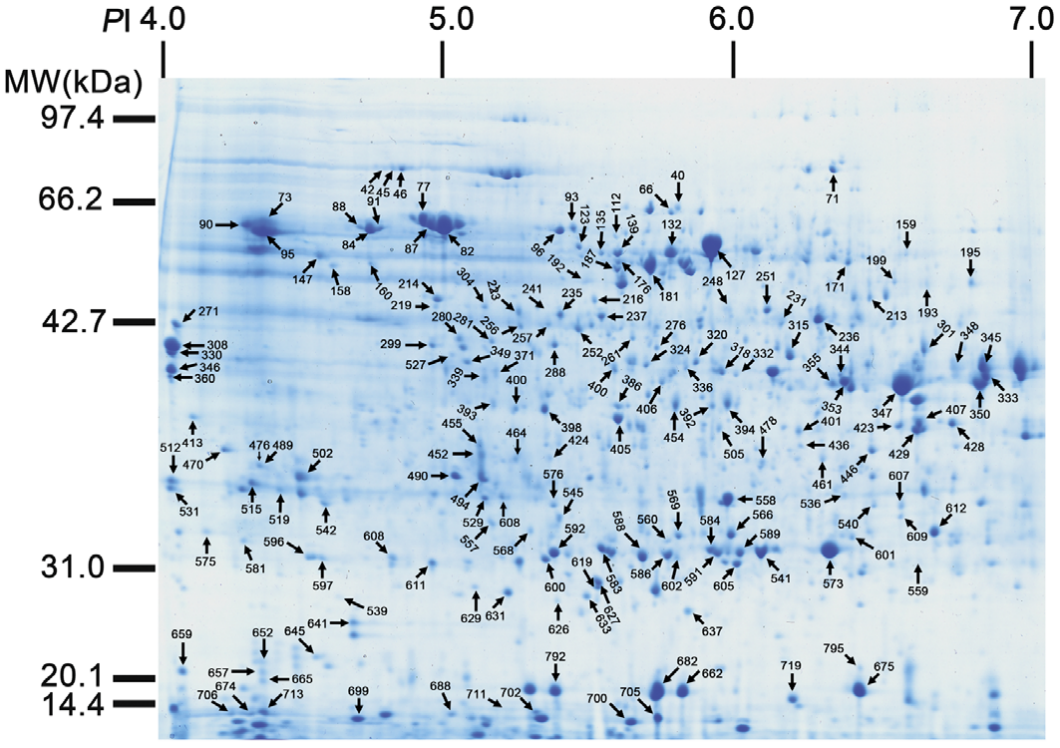
A representative image showing spot identification and localization of proteins from developing *Brassica campestri* L. seeds. Those spots indicated by arrows were excised from 2-D gels and analyzed by MALDI-TOF MS/MS. 209 proteins spots that have been identified are indexed as numbered, and the complete list of proteins is in the Table 1.

**Figure 3:**
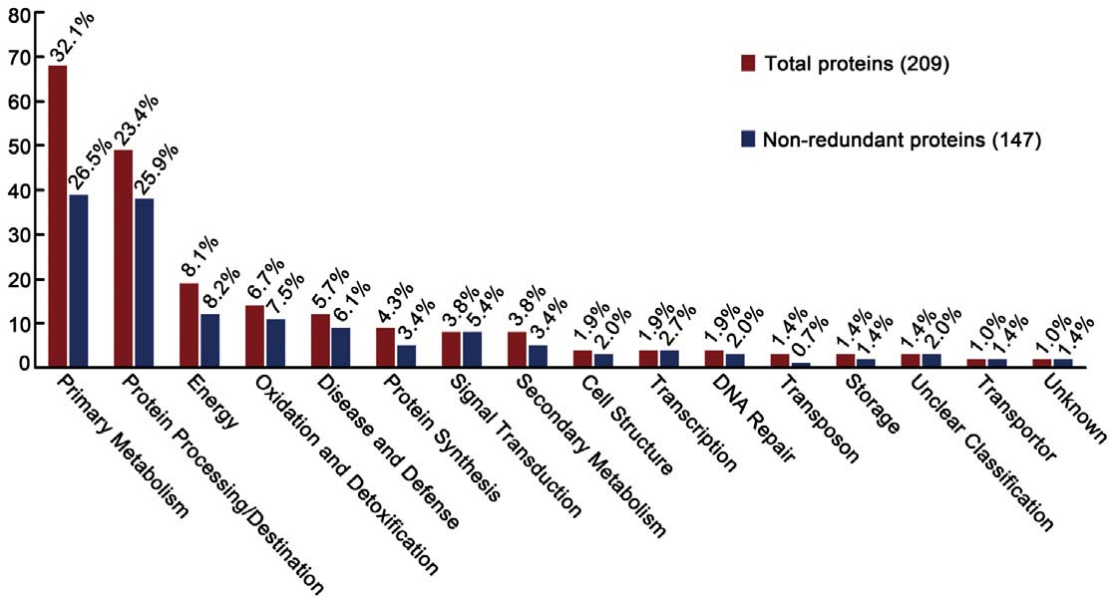
Functional classification of identified seed proteins of *Brassica campestri* L. The changed protein spots were identified by MALDI-TOF/TOF MS, and classified into 16 groups according to their functions using the NCBI da0ase. Out of a total of 209 identified proteins (red), 147 had nonredundant function (blue). The number above the bar indicates the proportion of each group of protein.

To select protein differentially accumulated over five developmental stages, their proteome profiles were compared using ImageMaster software and 260 spots with at least a two-fold change in statistical analysis (P≤0.05) in combination with manual validation and quantification. Then they were excised from the 2-DE gels and identified by MALDI-TOF/TOF-MS MASCOT and MASCOT database searching. Identities of a total of 209 proteins with altered accumulation were established (Table 1 and Figure 2). G/O analysis was carried out on the base of protein function and these proteins were classified into 16 groups, as depicted in Table 1, including primary metabolism, protein processing and destination, oxidation and detoxification, energy, transcription, protein synthesis, cell structure, signal transduction, defense, secondary metabolism, DNA repair and storage, suggesting these proteins should be involved in a wide range of cellular activities during seed development. Those proteins related to primary metabolism could be further classified into the TCA cycle, carbohydrate metabolism, fatty acid metabolism, nitrogen metabolism, amino acid metabolism, and others (Table 1). Importantly, these spots represented 147 non-redundant proteins, for example, five spots were identified to be enolase (127, 139, 248, 452, 132) and a total of 8 spots were found to be triosephosphate isomerase (591, 592, 557, 583, 588, 560, 573, 589), indicating some of the selected spots are isoforms or modified (Table 1). By calculating the relative proportions, it was found the most abundant proteins participated in the primary metabolism (32.1%), highlighting the dynamic requirement for the growing seed. The second group is related to protein processing/destination (23.4%) followed by those in energy production (8.1%), oxidation/and detoxification (6.7%), disease and defense (5.7%). The other processes these proteins got involved were protein synthesis (4.3%), signal transduction (3.8%), secondary metabolism (3.8%), cell structure (1.9%), transcription (1.9%), DNA repair (1.9%), transposon (1.4%), storage (1.4%), transporter (1.4%), unclear classification (1.0%) and unknown (1.0%) (Figure 3).

**Table 1:** List of total 209 seed proteins differentially accumulated over sequential seed development stages in *Brassica campestri* L.

Spot Number	Description	Species	ID	MW	PI	MOWSE Score	Relative Protein Abundance				
							Days After Pollination(DAP)				
**1. Primary metabolism**							10	16	20	25	35
**1.1 The TCA cycle**											
347	malate dehydrogenase	*Arabidopsis thaliana*	gi|15219721	35548.3	6.11	415	0.074	0.291	0.147	1.71	0.061
350	cytosolic malate dehydrogenase	*Arabidopsis thaliana*	gi|21593565	35639.4	7	161	0.132	0.152	0.107	0.457	0.027
355	malate dehydrogenase	*Arabidopsis thaliana*	gi|15219721	35548.3	6.11	234	1.438	0.493	0.083	0.077	0.044
428	mitochondrial NAD-dependent malate dehydrogenase	*Arabidopsis thaliana*	gi|21592905	35753.8	8.54	381	0.624	0.227	0.023	0.319	0.143
429	mitochondrial NAD-dependent malate dehydrogenase	*Arabidopsis thaliana*	gi|21592905	35753.8	8.54	358	0.119	0.088	0.046	0.2	0.059
423	chloroplast malate dehydrogenase	*Brassica napus*	gi|207667274	42292.2	8.51	773	0.795	0.237	0.031	0.324	1.279
446	chloroplast malate dehydrogenase	*Brassica rapa*	gi|207667274	42292.2	8.51	108	0	0	0.125	0.067	0.078
233	NADP-specific isocitrate dehydrogenase	*Arabidopsis thaliana*	gi|20260384	45703.1	6.13	232	0.099	0.159	0.398	0.117	0.075
371	pyruvate dehydrogenase E1 beta subunit	*Arabidopsis thaliana*	gi|21593379	43959	5.53	300	0.073	0.379	0.113	0.165	0.026
398	pyruvate dehydrogenase	*Arabidopsis thaliana*	gi|15241286	39151.1	5.67	382	0.189	1.84	0.079	0.085	0.793
400	pyruvate dehydrogenase	*Arabidopsis thaliana*	gi|15241286	39151.1	5.67	327	0.437	0.299	0.163	0.13	0.04
320	succinyl-CoA ligase	*Arabidopsis thaliana*	gi|15225353	45316.9	6.3	228	0.128	0.48	0.031	0.049	0.039
576	aconitase C-terminal domain-containing protien	*Arabidopsis thaliana*	gi|15224221	26773.5	6.33	422	0	0	0.18	0.5	0.64
611	aconitase C-terminal domain-containing protein	*Arabidopsis thaliana*	gi|15224221	26773.5	6.33	427	0	0	0.126	0.171	0.149
494	aconitase C-terminal domain-containing protein	*Arabidopsis thaliana*	gi|15224221	26773.5	6.33	442	0	0	0.054	0.208	0.11
**1.2 Carbohydrate metabolism**											
333	fructose-bisphosphate aldolase	*Arabidopsis thaliana*	gi|15231715	38515.9	6.05	190	0.059	0.358	0.133	0.037	0.038
345	fructose-bisphosphate aldolase	*Arabidopsis thaliana*	gi|15231715	38515.9	6.05	211	0.165	0.154	0.294	0.807	0.234
348	fructose-bisphosphate aldolase	*Arabidopsis thaliana*	gi|15231715	38515.9	6.05	128	0.314	0.146	0.114	0.26	0.073
71	2,3-bisphosphoglycerate-independent phosphoglycerate mutase	*Arabidopsis thaliana*	gi|21537260	60661.9	5.51	329	1.82	0.153	0.112	0.14	0.043
66	2,3-biphosphoglycerate-independent phosphoglycerate mutase	*Arabidopsis thaliana*	gi|18391066	60541.7	5.32	80	2.519	0.062	0.676	0.085	2.278
127	enolase	*Brassica napus*	gi|34597330	47346	5.46	247	0.237	0.155	0.116	2.24	0.126
139	enolase	*Brassica rapa*	gi|34597330	47346	5.46	154	0.56	0.324	0.461	0.393	0.051
248	enolase	*Brassica rapa*	gi|34597330	47346	5.46	216	0.075	0.723	0.12	0.127	0.082
452	enolase	*Brassica rapa*	gi|34597330	47346	5.46	138	0	0.01	0.07	0.52	0.065
132	Enolase (LOS2)	*Arabidopsis thaliana*	gi|15227987	47689.3	5.54	162	0.273	0.216	0.412	0.918	0.044
536	glyoxalase II	*Arabidopsis thaliana*	gi|1644427	27770	5.58	321	0	0	0.128	0.216	0.361
346	glyceraldehyde-3-phosphate dehydrogenase C	*Arabidopsis thaliana*	gi|21593240	36966.1	6.62	48	0.053	0.174	0.127	0.289	0.105
360	glyceraldehyde-3-phosphate dehydrogenase C2	*Arabidopsis thaliana*	gi|145323882	33884.5	6.67	40	0.356	0.144	0.125	0.101	0.061
332	phosphoglycerate kinase	*Arabidopsis thaliana*	gi|21536853	42121.4	5.49	345	0.053	0.565	0.234	0.068	0.037
336	phosphoglycerate kinase	*Arabidopsis thaliana*	gi|21536853	42121.4	5.49	630	0.089	0.673	0.23	0.131	0.063
591	triosephosphate isomerase	*Oryza sativa*	gi|553107	27588.3	6.6	222	0	0	0.09	0.356	1.095
592	triosephosphate isomerase	*Arabidopsis thaliana*	gi|414550	27138	5.24	188	0	0	0.243	0.187	0.182
557	triosphosphate isomerase-like protein type II	*Dimocarpus longan*	gi|262410515	27261.3	6.13	56	0	0	0.072	0.42	0.127
583	triosephosphate isomerase	*Arabidopsis thaliana*	gi|15226479	33325.1	7.67	287	0	0	0.234	0.352	0.055
588	cytosolic triosephosphate isomerase	*Arabidopsis thaliana*	gi|414550	27138	5.24	205	0	0	0.079	0.687	0.161
560	triosephosphate isomerase	*Oryza sativa*	gi|553107	27588.3	6.6	210	0	0	0.11	0.395	0.069
573	cytosolic triosephosphate isomerase	*Arabidopsis thaliana*	gi|414550	27138	5.24	150	0	0	0.064	1.404	0.091
589	cytosolic triosephosphate isomerase	*Arabidopsis thaliana*	gi|414550	27138	5.24	221	0	0	0.077	0.416	0.128
201	UTP-glucose-1-phosphate uridylytransferase	*Arabidopsis thaliana*	gi|15237947	51887.2	5.73	129	0.071	0.184	0.304	0.397	0.087
199	UTP-glucose-1-phosphate uridyly transferase	*Arabidopsis thaliana*	gi|15237947	51887.2	5.73	295	0.236	0.473	0.122	0.089	0.094
436	mannose 6-phoshpate reductase	*Arabidopsis thaliana*	gi|15226489	34988	6.16	141	0	0	0.011	0.084	0.123
135	alanine aminotransferase	*Arabidopsis thaliana*	gi|21954071	59496	5.91	101	0.123	0.206	0.426	0.151	0.097
413	beta-glucosidase, putative	*Ricinus communis*	gi|255542147	42630.9	5.58	58	0.138	0.367	0.301	1.008	0.072
251	UDP-D-apiose/UDP-D-xylose synthetase	*Gossypium hirsutum*	gi|211906520	43382.2	6.11	271	0.19	0.147	0.121	0.105	0.084
257	3-isopropylmalate dehydrogenase	*Arabidopsis thaliana*	gi|21553584	43272.7	5.8	61	0.187	0.757	0.436	0.156	0.14
261	3-isopropylmalate dehydrogenase	*Ricinus communis*	gi|255579212	43454.4	5.48	68	0.103	0.194	0.388	0.123	0.06
531	Trehalose-phosphatase family protein	*Oryza sativa*	gi|77555881	24791.3	5.58	47	0	0	0.114	0.285	0.425
**1.3 Fatty acid metabolism**											
405	enoyl-(acyl-carrier-protein) reductase	*Brassica napus*	gi|14422259	40683.8	8.53	375	0.51	0.963	0.657	0.495	0.15
386	enoyl reductase	*Brassica napus*	gi|1769966	40831.1	9.25	237	0.504	0.256	0.183	0.154	0.131
401	enoyl reductase	*Brassica napus*	gi|14422257	40865.2	9.27	193	0.43	0.388	0.052	0.148	0.151
193	3-ketoacyl-acyl carrier protein synthase1 (KAS1)	*Arabidopsis thaliana*	gi|79329956	44701.8	8.73	223	0.111	0.133	0.188	0.147	0.053
195	KAS1	*Arabidopsis thaliana*	gi|79329956	44701.8	8.73	364	0.182	0.21	0.325	0.286	0.018
670	3-ketoacyl-ACP dehydratase	*Brassica napus*	gi|14334124	24621.8	9.19	82	0	0	0.028	0.194	0.13
**1.4 Nitrogen metabolism**											
256	Glutamine synthetase	*Brassica napus*	gi|166406194	47315.8	5.84	515	0.291	1.074	0.159	0.101	0.029
315	glutamine synthetase	*Raphanus sativus*	gi|1526562	38494.2	5.93	383	0.255	0.636	0.357	0.24	0.109
**1.5 Amino acid metabolism**											
138	Imidazole glycerol-phosphate synthase	*Arabidopsis thaliana*	gi|222424719	64152.9	6.31	274	0.086	0.17	0.275	0.045	0.04
213	IAA amidohydrolase	*Arabidopsis thaliana*	gi|18129692	47986.3	5.81	244	0.1	0.201	0.096	0.085	0.037
235	Fumarylacetoacetase	*Arabidopsis thaliana*	gi|22329501	46066.1	5.31	117	1.112	0.609	0.181	0.136	0.052
237	fumarylacetoacetase	*Arabidopsis thaliana*	gi|22329501	46066.1	5.31	96	0.138	0.163	0.446	0.153	0.045
276	acetylomithine deacetylase	*Brassica oleracea*	gi|89257686	48003.3	5.45	401	0.158	0.593	0.247	0.178	0.132
581	indole-3-glycerol phosphate synthase	*Arabidopsis thaliana*	gi|21592587	44595.5	6.99	59	0	0	0.082	0.228	0.747
629	indole-3-glycerol phosphate synthase	*Arabidopsis thaliana*	gi|21592587	44595.5	6.99	215	0	0	0.045	0.092	0.154
271	indole-3-glycerol phosphate synthase	*Arabidopsis thaliana*	gi|21592587	44595.5	6.99	57	0.185	0.657	0.151	0.125	0.402
505	o-acetylserine lyase isoform A1 (OASA1)	*Arabidopsis thaliana*	gi|22331257	19625.2	7.71	80	0	0	0.109	0.097	0.062
**1.6 Others**											
668	D-galacturonic acid reductase 2	*Actinidia deliciosa*	gi|284437941	34701.8	6.67	53	0	0	0.062	0.39	0.3
236	MAT3 (methinonie adenosyltransferase 3(SAMhecheng)	*Arabidopsis thaliana*	gi|15228048	42470.6	5.76	272	0.134	0.615	0.125	0.087	0.058
231	SAM-2	*Arabidopsis thaliana*	gi|15234354	43227.8	5.67	554	0.063	0.77	0.187	0.144	0.048
537	GLYR1 (glyoxylate reductase 1)	*Arabidopsis thaliana*	gi|79313434	29500	6.22	42	0	0	0.25	0.5	0.24
**2. Protein synthesis**											
601	translation initiation factor eiF3	*Arabidopsis thaliana*	gi|17528988	66697.4	5.54	53	0	0	0.08	0.147	0.182
407	elongation factor	*Arabidopsis thaliana*	gi|23397287	74184.4	7.12	168	1.62	0.393	0.053	0.462	0.048
539	putative cysteinyl-tRNA synthetase	*Oryza sativa*	gi|22758381	59509.9	6.2	58	0	0	0.065	0.189	0.113
308	small ribosomal protein 4	*Encephalartos gratus*	gi|170516247	21815.8	10.54	44	0.221	0.173	0.056	1.258	0.045
681	small ribosomal protein 4	*Equisetum bogotense*	gi|16565378	21708	9.94	55	0	0	0.054	0.688	0.181
489	nascent polypeptide-associated complex subunit alpha-like protein 3	*Arabidopsis thaliana*	gi|240256288	22044	4.41	248	0	0	0.049	0.094	0.389
515	nascent polypeptide-associated complex subunit alpha-like protein 3	*Arabidopsis thaliana*	gi|240256288	22044	4.41	215	0	0	0.043	0.267	0.489
470	nascent polypeptide-associated complex subunit alpha-like protein 3	*Arabidopsis thaliana*	gi|240256288	22044	4.41	190	0	0	0.059	0.217	0.145
288	nascent polypeptide-associated complex subunit alpha-like protein 3	*Arabidopsis thaliana*	gi|240256288	22044	4.41	48	0.542	0.145	0.09	0.14	0.17
**3. Protein processing/destination**											
42	heat shock protein 70	*Arabidopsis thaliana*	gi|6746592	77058.6	5.13	295	0.248	0.224	0.499	0.304	0.223
46	chloroplast HSP70	*Cucumis sativus*	gi|124245039	75350	5.18	455	0.105	0.304	0.48	0.22	0.107
49	BIP2	*Arabidopsis thaliana*	gi|30693962	73515.9	5.11	428	0.542	1.33	0.438	0.277	0.176
53	BIP2	*Arabidopsis thaliana*	gi|30693962	73515.9	5.11	519	1.492	0.418	0.307	0.203	0.111
73	protein disulfide isomerase	*Brassica carinata*	gi|77999357	55706.2	133	133	0.049	0.227	0.361	0.588	0.038
77	ATPDIL1-2	*Arabidopsis thaliana*	gi|152223975	56329.4	4.9	87	0.763	0.458	0.388	0.226	0.043
82	ATPDIL1-2	*Arabidopsis thaliana*	gi|152223975	56329.4	4.9	120	0.241	0.34	0.546	1.26	0.123
87	ATPDIL1-1	*Arabidopsis thaliana*	gi|30687521	54125.4	4.97	115	1.687	0.96	0.47	0.324	0.23
90	ATPDIL1-1	*Arabidopsis thaliana*	gi|30687521	54125.4	4.97	320	0.715	0.084	0.298	0.541	0.064
91	CPN60b	*Arabidopsis thaliana*	gi|15222729	63769.6	6.21	129	0.483	0.117	0.094	0.085	0.704
112	BIP2	*Arabidopsis thaliana*	gi|30693962	73515.9	5.11	515	0.141	0.127	0.131	0.545	0.193
216	RPT3 (regulatory particle triple-A ATPase 3)	*Arabidopsis thaliana*	gi|15237159	45722.6	5.42	248	0.899	0.376	0.079	0.075	0.09
328	protein disulphide isomerase	*Brassica napus*	gi|45593261	25754.3	6.45	321	0.128	0.602	0.141	0.137	0.057
344	UNE5 (unfertilized embryo sac5)	*Arabidopsis thaliana*	gi|15226610	39472.4	5.8	111	0.156	0.692	0.101	0.105	0.079
353	UNE5 (unfertilized embryo sac5)-disulfite isomerase	*Arabidopsis thaliana*	gi|145331431	36514.8	5.49	178	2.46	0.234	0.151	0.864	0.039
455	PAF2	*Arabidopsis thaliana*	gi|15220151	30391	4.97	160	0	0	0.056	0.094	0.134
464	20S proteasome subunit PAF1	*Arabidopsis thaliana*	gi|3421092	30314.9	4.97	250	0	0	0.089	0.19	0.065
528	immunophilin	*Arabidopsis thaliana*	gi|1272408	17675.9	4.9	59	0	0	0.052	0.387	0.133
181	cytosol aminopeptidase	*Arabidopsis thaliana*	gi|15224101	54475	5.66	142	0.321	0.179	0.047	0.411	0.16
187	leucine aminopeptidase	*Ricinus communis*	gi|255576991	61171.6	8.09	72	0.132	0.265	0.059	0.173	0.075
548	cysteine proteinase inhibitor	*Brassica rapa*	gi|1256424	22930.6	5.95	221	0	0	0.21	0.6	0.22
559	PAC1-ubiquitin-dependent protein catabolic process	*Arabidopsis thaliana*	gi|15233268	27457.9	6.6	130	0	0	0.069	0.251	0.168
576	OUT-like cysteine protease family protein	*Arabidopsis thaliana*	gi|15223615	23410.6	4.98	49	0	0	0.23	0.42	0.64
592	PAE2-20S PROTEASOME ALPHA SUBUNIT E2	*Arabidopsis thaliana*	gi|15231824	25960.9	4.7	444	0	0	0.243	0.227	0.238
619	chaperonin 10	*Arabidopsis thaliana*	gi|3057150	26912.5	8.86	255	0	0	0.33	0.33	0.67
626	multicatalytic endopeptidase complex, proteasome precursor, beta subunit	*Arabidopsis thaliana*	gi|21592365	24016.1	5.7	544	0	0	0.037	0.089	0.127
527	peptidyl-prolyl cis-trans isomerase-like protein	*Arabidopsis thaliana*	gi|21554407	32917	7.66	41	0	0	0.012	0.214	0.115
635	peptidylprolyl isomerase ROC4	*Arabidopsis thaliana*	gi|21555831	28178.1	8.83	282	0	0	0.148	0.087	0.067
641	translationally-controlled tumor protein	*Zea mays*	gi|195605616	18730.4	4.53	69	0	0	0.107	0.203	0.234
645	VFB1 (VIER F-BOX PROTEINE 1)	*Arabidopsis thaliana*	gi|15220130	56459.9	8.93	54	0	0	0.057	0.06	0.316
45	CHLOROPLAST HEAT SHOCK PROTEIN 70-2	*Arabidopsis thaliana*	gi|15240578	76949.7	5.17	498	0.042	0.242	0.032	0.246	0.048
84	ATPDIL1-1 (PDI-LIKE 1-1)	*Arabidopsis thaliana*	gi|30687521	54125.4	4.97	207	0.067	0.514	0.096	0.75	0.122
93	chaperonin HSP60	*Arabidopsis thaliana*	gi|2924773	55218.9	5.3	160	0.138	0.375	0.087	0.09	0.325
96	chaperonin HSP60	*Arabidopsis thaliana*	gi|16221	61312.3	5.66	334	0.648	0.296	0.166	0.1	0.072
123	HSP60-3A	*Arabidopsis thaliana*	gi|18400195	60428.8	5.85	345	0.206	1.3	0.37	0.078	0.031
214	Tat binding protein like protein	*Brassica rapa*	gi|12697589	47448.3	4.91	586	0.462	0.273	0.165	0.104	0.045
542	senescence-associated cysteine protease	*Brassica oleracea*	gi|18141289	39309.3	5.47	180	0	0	0.047	0.174	0.175
566	PAA2 (20S PROTEASOME SUBUNIT PAA2)	*Arabidopsis thaliana*	gi|15224993	27332.8	5.75	363	0	0	0.125	0.148	0.225
569	PAA2 (20S PROTEASOME SUBUNIT PAA2)	*Arabidopsis thaliana*	gi|15224993	27332.8	5.75	384	0	0	0.06	0.165	0.164
586	PAB1 (PROTEASOME SUBUNIT PAB1)	*Arabidopsis thaliana*	gi|15219257	25685.3	5.53	249	0	0	0.093	0.805	0.232
590	chaperonin 10	*Arabidopsis thaliana*	gi|3057150	26912.5	8.86	158	0	0	0.06	0.543	0.118
597	endopeptidase	*Arabidopsis thaliana*	gi|15231824	26960.9	4.7	34	0	0	0.065	0.116	0.342
600	chaperonin 10	*Arabidopsis thaliana*	gi|3057150	26912.5	8.86	245	0	0	0.034	0.34	0.233
502	putative proteasome 20S beta1 subunit	*Brassica napus*	gi|41352683	18942.6	7.71	625	0	0	0.072	0.098	0.099
706	RPP3A (60S acidic ribosomal protein P3)	*Arabidopsis thaliana*	gi|15236029	11841.6	4.42	70	0	0	0.109	0.4	0.157
699	immunophilin	*Arabidopsis thaliana*	gi|1272408	17675.9	4.9	71	0	0	0.303	0.354	0.379
406	cyclase family protein	*Arabidopsis thaliana*	gi|18418598	29968.6	5.64	160	0.066	0.403	0.306	0.234	0.158
608	putative chloroplast nucleoid DNA-binding protein	*Arabidopsis thaliana*	gi|19424106	53186.5	5.26	98	0	0	0.268	0.245	0.27
675	peotidylprolyl isomerase ROC4	*Arabidopsis thaliana*	gi|21555831	28178.1	8.83	144	0	0	0.275	0.395	0
**4. Energy**											
131	Ribulose-1,5-bisphosphate carboxylase/oxygenase	*Arabidopsis thaliana*	gi|211573299	51767	6.04	140	0.134	0.629	0.145	0.741	0.041
219	Ribulose bisphosphate carboxylase activase	*Arabidopsis thaliana*	gi|30687999	48469.4	7.55	64	0.094	0.147	0.612	0.078	0.055
330	Ribulose-1,5-bisphosphate carboxylase	*Hevea brasiliensis*	gi|168997361	20732.2	8.44	61	0.288	0.89	0.808	0.249	0.045
135	mitochondrial F1 ATP synthase beta subunit	*Arabidopsis thaliana*	gi|17939849	63331.8	6.52	539	0.483	0.094	0.117	0.16	0.704
304	chloroplast rubisco activase	*Cucumis sativus*	gi|239837354	66841	4.49	315	0.134	0.169	0.054	0.345	1.247
490	oxygen-evolving complex (OEC)	*Arabidopsis thaliana*	gi|21593220	35136.6	5.55	543	0	0	0.09	1.44	0.876
609	adenosine kinase 2 (ADK2)	*Arabidopsis thaliana*	gi|15242717	37821.8	5.14	118	0	0	0.087	0.314	0.078
281	adenosine kinase 2 (ADK2)	*Arabidopsis thaliana*	gi|15242717	37821.8	5.14	465	0.147	0.275	0.052	0.247	0.27
612	oxygen-evolving complex (OEC)	*Arabidopsis thaliana*	gi|1076373	1433.7	9.71	68	0	0	0.303	0.103	0.09
633	PSBP-1	*Arabidopsis thaliana*	gi|186478207	23744.2	7.71	112	0	0	0.144	0.364	0.598
677	water-soluble chlorophyll protein	*Brassica oleracea*	gi|27530881	22720.9	7.83	66	0	0	0.059	0.337	0.09
682	water-soluble chlorophyll protein	*Brassica oleracea*	gi|27530881	22720.9	7.83	237	0	0	0.079	0.177	0.138
159	ATPase subunit 1	*Brassica napus*	gi|112253900	55096.8	6.01	275	0.083	0.233	0.551	0.224	0.034
529	PSBO2	*Arabidopsis thaliana*	gi|15230324	34997.7	5.92	371	0	0	0.244	0.244	0.269
631	PSBP-1	*Arabidopsis thaliana*	gi|186478207	23744.2	7.71	112	0	0	0.163	0.486	0.185
627	OEE2	*Arabidopsis thaliana*	gi|1076373	1433.7	9.71	96	0	0	0.469	0.613	0.762
280	adenosine kinase 2 (ADK2)	*Arabidopsis thaliana*	gi|15242717	37821.8	5.14	198	0.248	0.45	0.167	0.144	0.107
**5. Oxidation/Detoxification**											
324	peroxidase POA1	*Capsicum annuum*	gi|72534134	31852.9	8.43	76	0.245	0.493	0.881	0.067	0.106
339	peroxidase POA1	*Capsicum annuum*	gi|72534134	31852.9	8.43	79	0.064	0.126	0.608	0.17	0.05
393	peroxidase 27 (PER27)	*Arabidopsis thaliana*	gi|15232058	34927.9	9.16	65	0	0.8	0.07	0.1	0.04
705	glutathione peroxidase, putative	*Ricinus communis*	gi|255537447	18546.4	6.58	161	0	0	0.288	0.311	0.385
446	disulfide oxidoreductase, putative	*Ricinus communis*	gi|255575237	39320.1	8.2	243	0	0	0.216	0.113	0.091
719	copper/zinc superoxide dismutase	*Arabidopsis thaliana*	gi|3273753	22161.2	6.28	169	0	0	0.081	0.28	0
688	CCH (COPPER CHAPERONE)	*Arabidopsis thaliana*	gi|15228869	12962.6	4.91	112	0	0	0.189	0.379	1.86
605	GSH-dependent dehydroascorbate reductase 1	*Arabidopsis thaliana*	gi|21593056	23406.4	6	45	0	0	0.19	0.314	0.532
405	ascorbate peroxidase	*Brassica oleracea*	gi|18265379	27657.9	5.58	256	0.509	0.963	0.079	0.958	0.15
584	APX1 (ascorbate peroxidase 1)	*Arabidopsis thaliana*	gi|15223049	27543.8	5.72	224	0	0	0.389	0.244	0.15
575	cytochrome P450 monooxygenase	*Selaginella moellendorffii*	gi|157812615	56837.7	8.53	80	0	0	0.035	0.158	0.13
711	type 2 peroxiredoxin	*Arabidopsis thaliana*	gi|15231718	24668.9	9.12	250	0	0	0.214	0.834	0.148
702	type 2 peroxiredoxin	*Brassica rapa*	gi|4928472	17421.1	5.37	471	0	0	0.209	0.479	0.075
700	type 2 peroxiredoxin	*Brassica rapa*	gi|4928472	17421.1	5.37	289	0	0	0.255	0.762	0.047
**6. Secondary metabolism**											
645	(R)-limonene synthase, putative	*Ricinus communis*	gi|255587781	67886	5.87	90	0	0	0.044	0.063	0.316
652	(R)-limonene synthase, putative	*Ricinus communis*	gi|255587781	67886	5.87	65	0	0	0.045	0.382	0.155
657	(R)-limonene synthase, putative	*Ricinus communis*	gi|255587781	67886	5.87	80	0	0	0.04	0.633	0.61
671	(R)-limonene synthase, putative	*Ricinus communis*	gi|255587781	67886	5.87	65	0	0	0.062	0.552	0.221
301	CAD9 (CINNAMYL ALCOHOL DEHYDROGENASE 9)	*Arabidopsis thaliana*	gi|15235022	38908.6	6.21	155	0.417	0.2	0.917	0.244	0.063
545	caffeoyl-CoA 3-O-methyltransferase	*Brassica rapa*	gi|91694371	29005.8	5.21	221	0	0	0.033	0.128	0.143
454	zeta-carotene desaturase	*Zea mays*	gi|195654535	63050.5	7.53	79	0	0	0.219	0.136	0.1
713	phenylalanine amonnia lyase	*Populus trichocarpa*	gi|224109784	77644.7	5.83	60	0	0	0.124	0.682	0.052
**7. Transcription**											
223	reverse transcriptase	*Oryza sativa*	gi|62733278	161394.6	6.84	52	0.131	0.236	0.859	0.209	0.055
502	myb family transcription factor	*Arabidopsis thaliana*	gi|15233864	23097.5	6.92	53	0	0	0.072	0.098	0.099
607	transcription factor APF1	*Arabidopsis thaliana*	gi|13507025	30034.5	6.24	59	0	0	0.164	0.262	0.032
271	histone acetyltransferase	*Arabidopsis thaliana*	gi|18410098	63084.2	6.01	49	0.185	0.265	0.657	0.152	0.125
**8. Transportors**											
560	ATSAR1B (SECRETION-ASSOCIATED RAS 1 B)	*Arabidopsis thaliana*	gi|15223516	21972.4	6.52	64	0	0	0.11	0.395	0.069
476	AKT2/3 (arabidopsis potassium transport 2/3)	*Arabidopsis thaliana*	gi|18415864	91249.9	6.09	189	0	0	0.018	0.07	0.111
**9. Cell structure**											
241	actin-1	*Diospyros kaki*	gi|255291847	35616.1	5.57	390	0.121	0.268	0.17	0.101	0.083
519	kinesin motor domain containing protein	*Oryza sativa*	gi|77556349	316424.4	5	49	0	0	0.044	0.346	0.11
318	RGP4 (REVERSIBLY GLYCOSYLATED POLYPEPTIDE 4)	*Arabidopsis thaliana*	gi|15241258	41839	6.56	209	0.189	1.56	3.13	0.313	0.109
252	actin1	*Actinidia deliciosa*	gi|149938964	41637	5.31	625	0.589	0.228	0.504	0.276	0.118
**10. Signal transduction**											
541	annexin	*Arabidopsis thaliana*	gi|1429207	35757.2	5.2	62	0	0	0.057	0.874	0.161
88	calreticulin 2 (CRT2/1)	*Arabidopsis thaliana*	gi|15217459	48127.1	4.37	109	1.03	0.649	0.221	0.12	0.07
424	receptor protein kinase CLAVATA1	*Ricinus communis*	gi|255565085	105782.5	7.53	65	0.22	0.331	0.415	0.839	1.092
478	pas/lov protein B (PLPB)	*Arabidopsis thaliana*	gi|30678020	44660.4	6.68	80	0	0	0.237	0.871	0.345
574	phosphoprotein phosphatase 2A	*Arabidopsis thaliana*	gi|62321445	36064	5.32	76	0	0	0.049	0.7	0.109
674	calcineurin B-like protein 5 variant	*Oryza sativa*	gi|226731839	22525.4	5.48	59	0	0	0.125	0.394	0.599
95	calreticulin	*Arabidopsis thaliana*	gi|1009712	46554	4.37	57	0.226	0.24	0.45	2.3	0.97
699	calcium-dependent protein kinase	*Arabidopsis thaliana*	gi|15229002	64506.4	8.84	56	0	0	0.39	0.303	0.341
**11. Disease and defense**											
138	imidazoleglycerol-phosphate synthase	*Arabidopsis thaliana*	gi|15236905	64152.9	6.31	274	0.03	0.17	0.277	0.046	0.041
392	lesion initiation 2 (LIN2)	*Arabidopsis thaliana*	gi|240254000	43768.7	6.24	487	1.52	0.595	0.271	0.156	1.483
394	putative gag-pol precursor	*Oryza sativa*	gi|16905189	77155.9	8.74	78	0.402	0.136	0.205	0.335	0.1
512	metacaspase 2 (ATMC2)	*Arabidopsis thaliana*	gi|42567134	45781.1	5.37	56	0	0	0.083	0.48	0.056
568	NHO1 (nonhost resistance to *P.s.phaseolicola* 1)	*Arabidopsis thaliana*	gi|79321536	52449.7	6.11	64	0	0	0.143	0.432	2.25
665	NHO1 (nonhost resistance to *P.s.phaseolicola* 1)	*Arabidopsis thaliana*	gi|79321536	52449.7	6.11	79	0	0	0.07	0.446	0.209
792	putative blight resistance protein	*Oryza sativa*	gi|57899196	138160.7	6.78	83	0.093	0.591	0.114	0.082	0
586	At2G37660	*Arabidopsis thaliana*	gi|227204455	26279.8	5.29	250	0	0	0.093	0.806	0.169
662	heat stress-induced protein	*Brassica oleracea*	gi|3319646	23474.3	8.37	139	0	0	0.049	0.166	0.82
192	heat stress-induced protein	*Brassica oleracea*	gi|3319646	23474.3	8.37	224	0.529	0.213	0.17	0.115	0.051
659	nonhost resistance to P.s. phaseolicola1 (NHO1)	*Arabidopsis thaliana*	gi|79321536	52449.7	6.11	78	0	0	0.071	0.496	0.414
171	aldehyde dehydrogenase 2B4 (ALDH2B4)	*Arabidopsis thaliana*	gi|15228319	58552.1	7.11	275	0	0	0.177	0.317	0.057
**12. Transposon**							0	0.034	0.243	0.322	0.417
554	retrotransposon protein,putative	*Oryza sativa*	gi|110288898	112022.1	8.57	48	0	0	0.035	0.34	0.074
349	retrotransposon protein,putative	*Oryza sativa*	gi|77552111	225611	8.48	60	0	0.261	0.068	0.095	0.26
637	retrotransposon protein, putative, unclassified	*Oryza sativa*	gi|108709588	253746.6	7.62	65	0	0	0.212	0.117	0.065
**13. DNA repair**											
223	reverse transcriptase	*Oryza sativa*	gi|62733278	161394.6	6.84	90	0.131	0.146	0.859	0.209	0.055
158	DNA repair protein RAD23,putative	*Arabidopsis thaliana*	gi|15240922	40041.1	4.58	190	0.106	0.272	1.157	0.181	0.045
160	DNA repair protein RAD23,putative	*Arabidopsis thaliana*	gi|145334669	34690.5	4.85	175	0.135	0.459	0.189	0.167	0.258
147	RAD23-like protein	*Arabidopsis thaliana*	gi|30409726	36198.2	4.66	110	0.181	0.173	0.041	0.195	0.128
**14. Storage**											
540	cruciferin	*Brassica napus*	gi|167136	55973.2	6.84	198	0	0	0.036	0.145	0.083
558	napin	*Brassica napus*	gi|468018	20329	6.88	135	0	0	0.132	0.258	0.067
461	cruciferin	*Brassica napus*	gi|12751302	54349.6	8.13	138	0	0	0.216	0.245	0.222
**15. Unclear classification**											
795	stem-specific protein TSJT1	*Zea mays*	gi|226499994	27943.7	7.62	45	0	0.073	0.917	0.519	0.165
602	RNA recognition motif (RRM)-containing protein	*Arabidopsis thaliana*	gi|30693595	27731.4	8.94	33	0	0	0.075	0.087	0.2
299	acid phosphatase survival protein SurE	*Arabidopsis thaliana*	gi|18414342	34099.8	5.05	108	0.336	0.203	0.349	0.151	0.098
**16. Unknown**											
634	unkown	*Populus trichocarpa*	gi|118482805	43503.1	6.22	49	0	0.034	0.118	0.067	0.377
566	unknown	*Picea sitchensis*	gi|224284385	120613.4	5.89	55	0	0	0.125	0.255	0.04

### Protein Abundance Profiles of Each Functional Class

**Figure 4:**
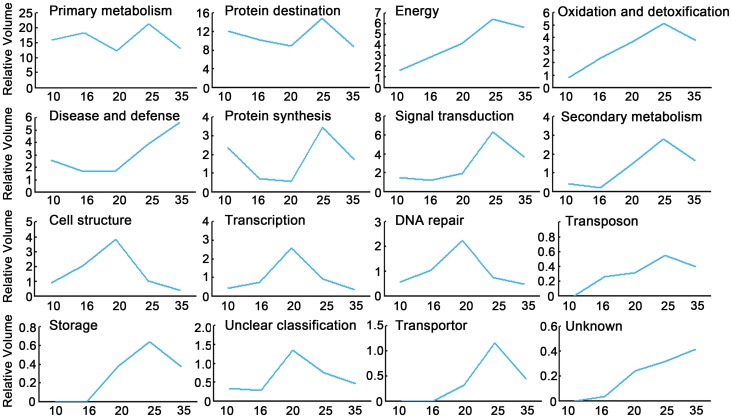
Composite protein abundance profiles of each functional categories. The combined accumulation profiles were calculated as the sum of expression value of all the proteins in each category (y axis) at each developmental stage (x axis).

To characterize global abundance kinetics of proteins involved in different processes, composite expression profiles were generated by summing protein abundance, expressed as relative volume [17], [30], [31], of each functional class over the five development stages. As shown in Figure 4, relative abundances of metabolic proteins fluctuated along the experimental period, reflecting different metabolic activity during the embryo maturation. Abundance of those responsible for protein synthesis, destination and second metabolism decreased during early seed growth, but increased and reached the top at the 25 DAP before a second reduction. Disease- and defense-related proteins were highly abundant at the late stage of seed development, and those involved in energy production, oxidation and detoxification, signal transduction, transposition, storage and transportation shared very similar patterns which increased and reached the summit at 25 DAP, whereas proteins related to cell structure, transcription, DNA repair and continued to accumulate and had the highest abundance at 20 DAP (Figure 4). Generally, it’s very interesting to find most of the protein groups possessed relatively higher abundance at a stage from 20 DAP to 25 DAP, reflecting extensive cellular activities during the processes of seed development.

### Hierarchical Clustering Analyses of Seed Proteins

**Figure 5:**
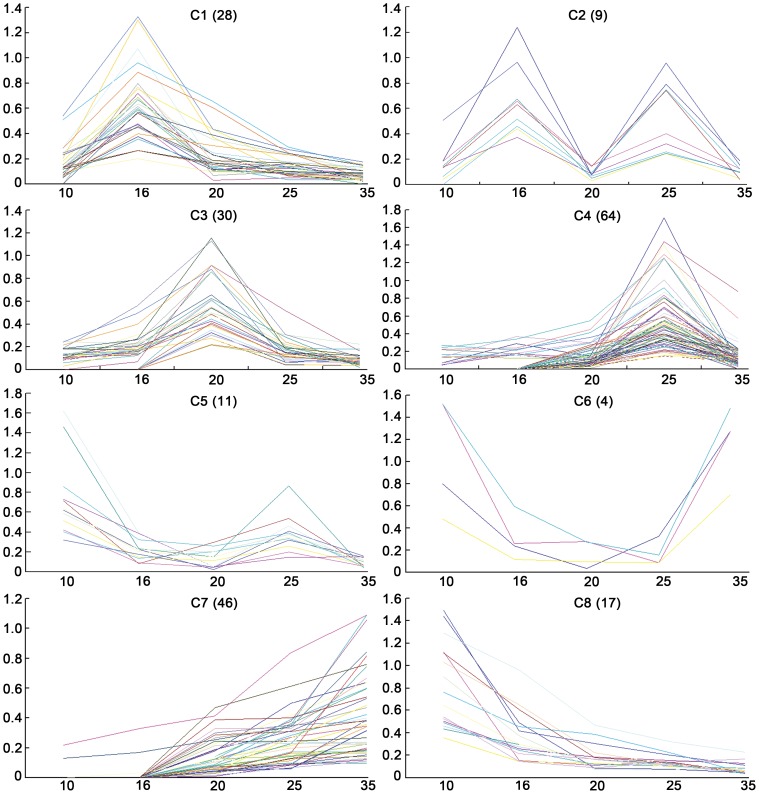
Abundance profiles of eight hierarchical clusters (c1–c8). The profiles were established by the protein expression value (vertical axis) at each developmental stage (horizontal axis). The expression level of the differentially accumulated proteins within each cluster is shown. Each colored line represents a protein identity.

To further improve the understanding of the identified proteins, abundance profiles were analyzed by hierarchical clustering. Finally, we generated a total of eight cluster groups (c1, c2, c3, c4, c5, c6, c7 and c8) that displayed similar dynamics (Table 2 and Figure 5), suggesting complicated regulatory patterns of these identified proteins during the seed development. The largest group contained 64 proteins (c4), expression of which increased from the early stage of seed development and reached the top at the 25 DAP but decreased at the late stage (35 DAP). The second group included 46 proteins (c7), and most of them were not detected until 16 DAP and were highly accumulated even at 35 DAP, different from those in the group of c1. The smallest cluster, c6, had only four proteins which displayed U-type expression profiles (Figure 5). Clusters c1 and c3 consisted of 28 and 30 proteins, which had the highest abundance at 16 DAP and 20 DAP, respectively. Seventeen proteins were grouped into the c8, and their abundance remained reducing along the seed growth. Notably, most of the proteins involved in primary metabolism, energy production, protein destination and oxidation were included into the c4 group (Figure 5 and Table 2), suggesting these cellular activities are essential for the early-stage seed development.

**Table 2:** Hierarchical clusters of differentially accumulated seed proteins and distribution of the proteins belonging to each category in different clusters.

Categories	C1	C2	C3	C4	C5	C6	C7	C8	Total
1. Primary Metabolism	15	2	10	16	5	2	10	7	67
2. Protein processing/destination	6	3	3	13	3	1	14	6	49
3. Energy	2	2	3	6	0	0	4	0	17
4. Oxidation and Detoxification	2	1	3	5	0	0	3	0	14
5. Disease and Defense	1	0	1	5	1	1	2	1	12
6. Protein synthesis	0	0	0	4	1	0	3	1	9
7. Signal transduction	0	0	0	4	0	0	3	1	8
8. Secondary metabolism	0	0	2	4	0	0	2	0	8
9. Cell structure	1	0	1	1	1	0	0	0	4
10. Transcription	0	0	2	1	0	0	1	0	4
11. DNA repair	1	0	3	0	0	0	0	0	4
12. Transposon	0	1	1	1	0	0	0	0	3
13. Storage	0	0	0	2	0	0	1	0	3
14. Transporter	0	0	0	1	0	0	1	0	2
15. Unclear classification	0	0	1	0	0	0	1	1	3
16. Unknown	0	0	0	1	0	0	1	0	2
Total	28	9	30	64	11	4	46	17	209

## Discussion

### Proteins Associated with Metabolism and Protein Renewal are Prevalent in the Developing Seed

Currently, a large number of proteomic studies have been carried out in different species to understand seed development [11], [32]. Most of these studies, both in embryo-dominant seeds and endosperm dominant seeds, identify the largest group of proteins involved in metabolism, which is consistent with the rapid and complicated metabolic changes during seed development [12]. Our analysis revealed similar character that a proportion of 32.1% total identified proteins participated in primary metabolism (Figure 3). For example, for the enzymes involved in the glycolytic pathway, 7 of them were identified as 15 protein spots. Besides, five enzymes of TCA pathway and three enzymes in lipid biosynthesis were identified (Table 1). An obvious pattern shared by these enzymes is their accumulation remained increasing over five stages of seed development (Figure 5), indicating these metabolic pathways were increasingly required for the seed development. Many proteins with altered expression in our analysis were related to other metabolic events, like amino acid metabolism. In addition, proteins associating with energy and metabolism, defense, oxidation/detoxification were prevalent in the developing seed (Table 1). Interestingly, one transcript study on Arabidopsis embryo development indicates that transition from globular to torpedo stage is associated with up-regulation of genes involved in energetics and metabolism [33], which is consistent to our proteomic study. Abundance of these proteins probably suggests that their activities defined the basal requirement during seed development.

Our data revealed 23.4% total proteins (25.9% of nonredundant proteins) were involved in protein processing and destination (Figure 3). They were those molecular chaperons that helped protein folding of newly synthesized proteins (spot 42, 53, 73), those isomerases that functioned in changing protein conformation (spot 353, 328, 406 and 675), the ubiquitin proteasome group including 20S, 26S proteasome subunits (spot 91, 464, 592, and 569), and those proteases (spot 328, 576 and 406), suggesting important protein turnover and rearrangements during seed development. Ubiquitination-mediated degradation pathway plays an important role in various aspects of plant growth and development [34]. Polyubiquitinylation of substrates is achieved through the action of three enzymes: E1, ubiquitin-activating enzyme, E2, ubiquitin-conjugating enzyme, and E3, ubiquitin ligase that determines the specificity of the substrate. The modified protein is then processed by the 26S proteasome, which consists of a core 20S protease capped at each of its ends by a regulatory 19S complex [35]. In our analysis, four isoforms of E1 and eight proteasome components were observed (Table 1). Folding of nascent polypeptides into functional proteins is controlled by a number of molecular chaperones and protein-folding catalysts. Our analysis revealed 6 different isoforms of protein disulphide isomerases, an endoplasmic reticulum-located protein that catalyzes the formation, isomerization, and reduction/oxidation of disulfide bonds [36]. Seven chaperonins or chaperones were also observed, including the plant homolog of the immunoglobulin heavy-chain binding protein (BiP), which is an endoplasmic reticulum- localized member of the heat shock 70 family. BiP has been proposed to play a role in protein body assembly within the endoplasmic reticulum [37], [38].

These proteins displayed different accumulation patterns in the process of seed development. For example, spot 73 was identified as a protein disulfide isomerase that continued to accumulate and reached the highest at 20 DAP. Consistent with this in the transcript level, our gene expression analysis also revealed disulfide isomerase can be detected at the late stage of embryogenesis [39]. Plant cysteine proteases are important for organ senescence, plant defense and nutrient mobilization during seed germination [40], and previous studies reveal cysteine proteinases are up-regulated in various senescing plants, such as *Arabidopsis, B. napus*, and *Nicotiana tabacum* [41]. In this study, we identified spot 542 as senescence-associated cysteine protease, and spot 576 as another cysteine proteinase that increased its abundance all over the five stages (Figure 5), suggesting that cysteine proteinase also played an important role in maturation and senescence of seed growth. Altered accumulation of these proteins indicated active protein production and elimination occurred in the process of seed development, which might serve as a monitoring mechanism over those intricate processes of metabolism and energy production. It’s also highly likely that the accumulation of these proteins may be used during rapid cell division and cell structure construction. Despite of these, preponderance of these proteins seemed to be particular of our study, because few of previous reports has indicated so many proteins with similar function [13], [42]–[44], which make us underestimate the importance of protein self-renewal. Therefore, protein renewal could be an essential regulatory mechanism for seed development.

### Carbon Assimilation During Seed Development

**Figure 6:**
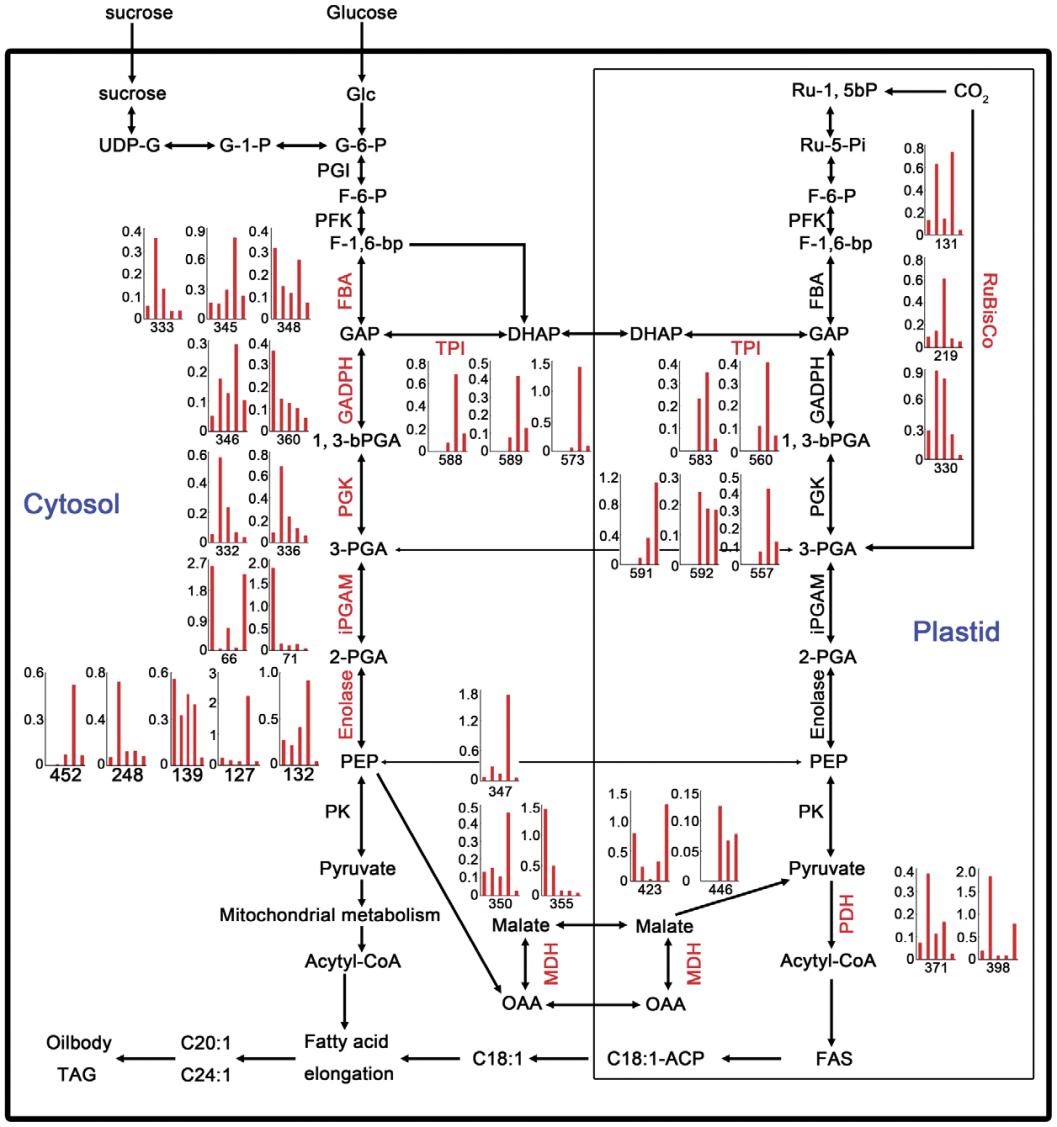
Characterization of central carbon metabolism in developing seed of *Brassica campestri* L. Proteins involved in sugar metabolism that provide carbon sources for biosynthetic pathways are shown (red). The × axis shows each of the five stages and the y axis shows the relative volume of identified proteins. Abbreviations for metabolites: UDP-G, UDP-Glc; G-1-P, Glc 1 phosphate; G-6-P, Glc 6 phosphate; F-6-P, Fru 6 phosphate; F-1,6-bP, Fru 1,6 bis phosphate; GAP, glyceraldehyde 3-P; DHAP, dihydroxyacetone phosphate; 1,3-bPGA, 1,3 bis phosphoglyceric acid; 3-PGA, 3 phosphoglyceric acid; 2-PGA, 2 phosphoglyceric acid; PEP, phosphoenolpyruvate. Abbreviations for enzymes: PGI, phosphoglucose isomerase; PFK, pyrophosphate-dependent phosphofructokinase; FBA, Fru bisphosphate aldolase; GAPDH, glyceraldehyde 3-P dehydrogenase; TPI, triose-phosphate isomerase; PGK, phosphoglyceratekinase; iPGAM, 2,3-bisphosphoglycerate-independent phosphoglycerate mutase; PRK, phosphoribulokinase; Rubisco, ribulose-1,5-bisphosphate carboxylase; PK, pyruvate kinase; PDH, pyruvate dehydrogenase; MDH, malate dehydrogenase.

The developing oilseeds take up sugars and amino acids from the surrounding endosome liquid and synthesize large quantities of triacylglycerol storage proteins. Previous work characterizes carbon assimilation during seed filling in *Brassica napus* and castor, both of which are oil plants [17], [45]. It’s interesting to examine this important metabolism pathway in the seed development. It has been demonstrated that glycolysis supplies most carbon to fatty acid synthesis (FAS) in rapeseed developing embryos in culture [46], suggesting glycolysis is essential for carbon assimilation in the developing seeds, but relatively little is known about its regulation and control, and due to the parallel pathways operated in both the cytosol and plastids, it become more complex in plants [47], [48]. Our study revealed a large number of protein spots corresponding to numerous different glycolytic enzymes both in the cytosol and plastids. FBA catalyzes the aldol cleavage of Fru-1,6-bisP to glyceraldehydes-3-P (GAP) and dihydroxyacetone phosphate(DHAP), and three cytosolic FBA spots were identified in this study (spot 333, 345 and 348) and their expression profiles are different from each other during the development (Figure 6). Triose-P isomerase (TPI) catalyzes the interconversion of GAP and DHAP and eight TPI (spot 588, 589, 573, 583, 560, 591, 592 and 557), with three in cytosol and five in plastid, were identified (Figure 6). Interestingly, their accumulation was relatively higher at the late stages of seed development. Glyceraldehyde 3-P dehydrogenase (GAPDH) reversibly catalyzes the conversion of GAP into 1,3-bisPGA, and two cytosolic spots (spot 346 and 360) of GAPDH with different expression were identified (Figure 6). Two cytosolic forms of 2,3-bisphosphoglycerate-independent phosphoglyceratemutase (iPGAM) (spot 66 and 71) and two phosphoglycerate kinase spots (PGK) (spot 332 and 336) were separately identified (Figure 6). In addition, five enolases (spot 452, 248, 139, 127 and 132) and malate dehydrogenase (MDH) (spot 347, 350, 355, 423 and 446) and most of them are cytosolic (Figure 6). It is notable their expression peaked at different seed growth stages, suggesting their importance at different development time. Consistent with a proteomic analysis of seed filling in *B. napus* which suggests that sugar mobilization from glucose to coenzyme A and its acyl derivative is a collaboration between the cytosol and plastids, and temporal control of enzymes and pathways extends beyond transcription [17], the detection of multiple isoelectric species for cytosolic and plastidial glycolytic enzymes indicated balanced coordination between cytosolic and plastidial glycolysis during seed development.

### Possible Seed Development-specific Proteins Indicated by This Study

In our analysis, four spots were identified to be proteins related to cell structure, taking a proportion of only 1.9% of total proteins (Figure 3). This is obviously lower than previous studies on seed development in Arabidopsis [16] or *B. napus* [18], which often identifies similar proteins of about 20%. Protein level of spot 318, RGP4, continued increasing from the beginning of seed development until at 16 DAP, and could be hardly detected at the late stage (Figure 5), suggesting it might be a novel protein associating with seed development. This is consistent with recent report that expression of RGP4 was restricted in seed and important for development [49]. Such proteins as Actin1 (spot 241, 252) has been found to be highly dynamic in nearly every development stages of seed development, highlighting their importance. Despite these studies use different materials of different development stages, the relative low proportion of proteins contributing to cell structure in this report may suggest another novel character specific to *B. campestris* seed development.

Our analysis identified only two storage proteins: napin (spot 558) and cruciferin (spot 461 and 540 ), which are the two major storage proteins in rape seed (*B. napus*), and constitute 20% and 60% of the total protein in mature seeds [50]. As has been reported that their biological synthesis begins early from the expansion phase of embryo development [51]. Consistent with their roles as “molecular marker” of late embryogenesis, both proteins were found to continually accumulate over the five embryo development stages and reach the highest level in the last stage (Figure 5). In our parallel gene expression analysis, *napin* gene was found to be up-regulated obviously during embryogenesis and expressed highly only in late embryo stage, but it was not detected in the globular embryo stage [39]. Interestingly, few of previous embryo-related proteomic studies have reported storage protein of napin, in contrast to that cruciferin is frequently detected. Expression of a novel protein, AKT2/3 (spot 476), increased in the early stage of seed development and began to decrease after 20 DAP (Figure 5). In Arabidopsis, AKT2/3 encodes photosynthate- and light-dependent inward rectifying potassium channel with unique gating properties that are regulated by phosphorylation [52], [53]. Therefore, identification of AKT2/3 suggested its novel role in seed development. Another interesting finding comes from an unclassified protein TSJT1 (spot 795), which has been indicated as stem-specific and found in gene chip data [54]. During seed development, it was very highly expressed early at 10 DAP, but after 20 DAP, its protein significantly decreased (Table 2 and Figure 5), therefore, our analysis indicates it may be important for early seed development, which remains to be determined by further experiment.

An investigation on seed development should significantly enrich our knowledge on the molecular and physiological events in whole seed growth process. In this study, we explored the protein dynamics over five stages during *B. campestri* seed development using a proteomic approach. A total of 209 proteins were identified by mass spectrometry to be differentially seed development and they could be classified into 16 functional groups. It was found that proteins participating in metabolism, energy production, oxidation/detoxification as well as stress/defense were highly dynamic in abundance. However, expressed during functional assignment of these altered proteins uncovers unexpected abundance of proteins related to protein processing and destination, highlighting the importance of protein renewal in seed development, and proportion of those associated to cell structure was rather low compared to previous proteomic analysis of seed development. Our study provides important information to better understanding the seed development in oil plant.

## Materials and Methods

### Plant Materials and Sample Collection

*Brassica campestri* L. (cv. Jianghuangzhong) plants were grown in soil-based compost under natural conditions (Wuhan, China). Before flowering, nylon nettings were used to prevent pollen contamination. For sampling seeds in different developmental stages, flowers were tagged immediately after opening of buds, and development of seeds was monitored by checking the embryos under a dissecting microscope. Harvesting the developing seeds was performed at precisely 10, 16, 20, 25 and 35 days after pollination (DAP) when their embryos were at the globular embryo stage, heart stage, torpedo stage, bended-cotyledon stage and C-shaped mature embryo, respectively. Five grams of seeds in each stage were sampled. Then they were frozen in liquid nitrogen and stored at −80°C for use.

### Protein Extraction

One gram of seed samples were grounded with mortar and pestle into fine powder in liquid nitrogen, then they were immediately homogenized with ice-cold extraction buffer (8 M Urea, 2 M Thiourea, 4% w/v CHAPS, 40 mM Tris-HCl, pH 8.0) containing protease inhibitors (1 mM PMSF, 10 mM DTT). The supernatant was collected by centrifugation at 20000 g for 30 min at 4°C. Then the pellet was resuspended in ice-cold lysis buffer and centrifuged as described above. After the oil above the supernatant was removed, proteins in the supernatant were precipitated with five volumes of ice-cold trichloroacetic acid–acetone (12.5% trichloroacetic acid in 100% acetone) at −20°C for 2 h and then collected by centrifugation at 20000 g for 30 min. The pellet proteins were resuspended in 80% ice-cold acetone containing 20 mM DTT and centrifuged as above for two times before they were dried by vacuum. The obtained proteins were dissolved in lysis buffer (8 M urea, 4% CHAPS, 10 mM DTT, and 2% pharmalyte 4–7) at room temperature, then vortexed vigorously and centrifuged. The final supernatants were transferred to fresh tubes. The protein concentration was quantified according to the Bradford method [55] using UV-2000 UV-visible spectrophotometry (UNICO) with bovine serum albumin (BSA) as the protein concentration standard. The final protein samples were stored at −70°C for two-dimensional gel electrophoresis (2-DE).

### Two-dimensional Electrophoresis (2-DE)

For protein identification, one milligram protein samples in 450 µl rehydration solution containing 8 M Urea, 4% (w/v) CHAPS, 0.5% (v/v) IPG buffer (pH 4–7) (GE Healthcare), 20 mM DTT and 0.002% w/v bromphenol blue were loaded onto 24 cm IPG strips (pH 4–7) (GE Healthcare) after brief sonication and centrifugation. Isoelectric focusing (IEF) was performed at 100 V (1 h), 300 V (1 h), 500 V (1 h), 1000V (1 h) and 8000V (12 h) using the Ettan™ IPGphor III ™ Isoelectric Focusing System (GE Healthcare). Before the second dimension, strips were equilibrated in the buffer containing 6 M urea, 75 mM Tris-HCl (pH 8.8), 30% (v/v) glycerol, 2% (v/v) SDS, 0.002% (w/v) bromophenol blue and 10 mg/ml DTT for 15 min, then in 25 mg/ml iodoacetamide (15 min) for a second equilibration step. For the second dimension, proteins were separated on 12.5% SDS acrylamide gels (26×20×0.1 cm) at 2.5 W/gel for 45 min, then at 15W/gel for 5 h using the Ettan DALT six System (GE Healthcare). For each development stage, three gels were run and then stained with Coomassie brilliant blue dye (CBB R-250) [56] and the experiment was repeated three times with similar results.

### Imaging and Statistical Analysis

2-DE gels stained by CBB were scanned at a resolution of 300 dpi and 16-bit pixel depth and then analyzed by ImageMaster 2-D Platinum 6.0 software (GE Healthcare) according to protocols provided by the manufacturer. After automatic spot detection, manual spot editing was carried out. Spots matching in at least two out of three gels for each protein extraction were considered as reproducible spots and included in the synthetic 2-DE gel images. Spot matching was further confirmed by visual inspection. To determine the differences in protein abundance across distinct 2-DE gels, the normalized/relative protein spot volume (area multiplied by stain intensity) calculated by the ImageMaster 2-D Platinum 6.0 software (GE Healthcare) was used as the parameter, and protein spots with changes more than two folds (P<0.05) by statistic analysis were considered as differentially accumulated. Then, significantly dynamic spots (P<0.05) were re-examined by eye detection to include only the most obviously varying spots over different stages for further study.

### Protein Identification by MALDI-TOF/TOF MS

Dynamically accumulated protein spots among five developmental stages were manually excised from 2-D identification gels and digested with trypsin (Promega). Each dried peptide mixture was dissolved into a volume of 50% ACN/0.1% TFA according to its relative abundance in the gel. Then the salts and detergents were removed using Millipore C18 ZipTips (Millipore). Bound peptides were eluted from ZipTip with approximately 3 µl 60% methanol/3% formic acid. 0.5 µl sample solution or calibration standard was then mixed with equal volume of CHCA (a-cyano-4-hydroxycinnamic acid) matrix (10 mg/ml CHCA in 50% ACN/0.1% TFA) and spotted onto a freshly cleaned target plate. After air drying, the crystallized spots were analyzed by MALDI-TOF/TOF (4800 Plus Analyzer, Applied Biosystems). Parent mass peaks were scanned in 1000 laser shots with a mass range of 800∼4000 Da after calibration. The minimum signal to noise ratio was 10. Five parent mass peaks with most intensity were picked out for tandem TOF/TOF analysis, each with 1500 laser shots. The searching parameters were set as follows: carbamidomethylation (C) and oxidation (M) as variable modifications, up to one missed cleavage, precursor ion tolerance at 200 ppm, and fragment ion tolerance at 0.3 Da and peptide charge of 1+. Protein hits with protein scores C. I.% (confident identification percentage, based on combined mass and mass/mass spectra) over 95 were reserved. Most identified proteins also have total ion score C. I.% (based on mass/mass spectra) over 95. Spectra combined mass and mass/mass were searched against an NCBInr protein database, taxonomy Viridiplantae (Green Plants) by GPS Explorer™ Workstation (Applied Biosystems).

### Hierarchical Cluster Analysis

Gene Cluster 3.0/TreeView software was used to do the clustering based on the mean relative volume of each protein spot. Clustering is based on visual inspection of relative similarities or differences between different cluster ranges and the number of clusters was chosen when the dynamics of functional categories between clusters possesses the most significant difference.
